# In-flight angina pectoris; an unusual presentation

**DOI:** 10.1186/s12872-018-0797-1

**Published:** 2018-04-05

**Authors:** Firas Al-Janabi, Regina Mammen, Grigoris Karamasis, John Davies, Thomas Keeble

**Affiliations:** 1 Essex Cardiothoracic Centre, Basildon Hospital, Nethermayne, Basildon, Essex, SS16 5NL UK; 2 Anglia Ruskin University, Bishop Hall Lane, Chelmsford, Essex, CM1 1SQ UK; 3 St Bartholomewʼs Hospital, W Smithfield, London, EC1A 7BE UK; 4 Southend General Hospital, Prittlewell Chase, Westcliff-on-Sea, SS0 0RY UK

**Keywords:** Angina, Flight, Coronary artery disease, Pressure wire, Fractional flow reserve

## Abstract

**Background:**

An unusual case of typical angina which occurred on a long haul flight is presented. This case is notable as this was the index presentation, with no previous symptoms prior to this. Physiological changes at altitude can be marked, and include hypoxia, tachycardia and an increase in cardiac output. These changes were enough to expose underlying angina in our patient.

**Case presentation:**

A 68 year old man presented with typical cardiac chest pain on a long haul flight. His symptoms first started 10-15 min after take-off and resolved on landing. This was his index presentation, and there were no similar symptoms in the past. Background history included hypercholesterolaemia and benign prostatic hypertrophy only. He led a rather sedentary lifestyle.

A CT coronary angiogram showed significant disease in the proximal left anterior descending artery and proximal right coronary artery. He went on to have a coronary angiogram with invasive physiological measurements, which determined both lesions were physiologically significant. Both arteries were treated with drug eluting stents.

Since treatment, he once again embarked on a long haul flight, and was completely asymptomatic.

**Conclusion:**

The presentation of symptoms in this individual was rather unusual, but clearly caused by significant coronary artery disease. Potentially his sedentary lifestyle was not enough in day-to-day activities to promote anginal symptoms. When his cardiovascular system was physiologically stressed during flight, brought about by hypoxia, raised sympathetic tone and increased cardiac output, symptoms emerged. In turn, when landing, with atmospheric conditions normalised, physiological stress was removed, and symptoms resolved. Clinically therefore, one should not exclude symptoms that occur with differing physiological states, such as stress and altitude, as they are also potential triggers for myocardial ischaemia, despite absence of day-to-day symptoms.

## Background

The physiological effects of high altitude on the cardio-respiratory system are well-known. Exposure to high altitudes can exacerbate the symptoms of underlying disease, depending on the altitude and length of exposure. Most of the reported in-flight medical emergencies occur in people with pre-existing medical conditions, with exacerbation of respiratory conditions being the commonest complaint, followed by angina [1]. However, occasionally new symptoms arise in otherwise healthy individuals. The commonest medical emergencies encountered in-flight are syncope and gastro-intestinal disorders [1, 2].

We present the case of a 68 year old gentleman with no significant past medical history who developed new onset of angina whilst on a long haul flight, with symptoms occurring at cruising altitude and resolving on landing. He had no prior symptoms, making his presentation unusual.

## Case presentation

A 68 year old gentleman was referred to the cardiology clinic due to an episode of chest pain which occurred whilst travelling from the UK to Australia via Singapore. He developed chest tightness after take-off in the UK which persisted continuously for the entire duration of the flight until it landed in Singapore on transit. When the flight touched down on ground, he noticed that the tightness had completely disappeared. It recurred after take-off and once again, persisted throughout the journey from Singapore to Australia. The pain was cardiac in nature. He has never smoked. A chest x-ray and a CT pulmonary angiogram done previously had not revealed any lung parenchymal disease. His cholesterol levels were 5.3 with raised low density lipoprotein (LDL) of 3.5. There was no significant past medical history except for benign prostatic hypertrophy for which he took Finasteride and Tamsulosin. Physical examination was unremarkable.

**Fig. 1:**
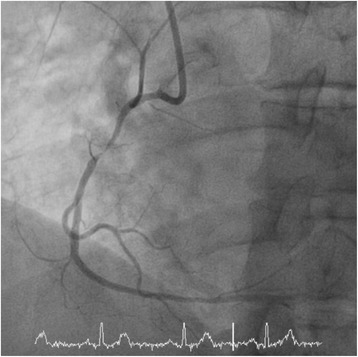
Invasive angiography demonstrating a severe proximal right coronary artery (RCA) lesion. On invasive physiological assessment, this was deemed a flow limiting stenosis and treated with a drug eluting stent

**Fig. 2:**
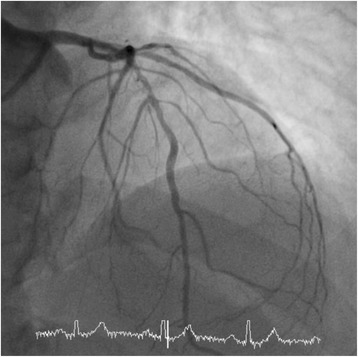
Invasive angiography demonstrating a long lesion in the proximal left anterior descending artery (LAD). Once again, on invasive physiological assessment, this was deemed flow limiting, and treated with a drug eluting stent

His resting electrocardiogram (ECG) revealed a normal sinus rhythm with normal cardiac intervals. His clinical examination was normal. Blood pressure in clinic was raised at 165/112 mmHg, however, a subsequent 24 h blood pressure monitor only revealed a mildly raised mean nocturnal blood pressure of 137/87 mmHg. Initially a CT coronary angiogram was organised due to the unusual presentation. This revealed significant stenoses in the proximal to mid left anterior descending artery (LAD) and the proximal right coronary artery (RCA) with a 50% stenosis in the circumflex and obtuse marginal artery. An invasive coronary angiogram confirmed critical proximal to mid RCA stenosis (Fig. 1) and a long segment of proximal LAD stenosis (Fig. 2).

**Fig. 3:**
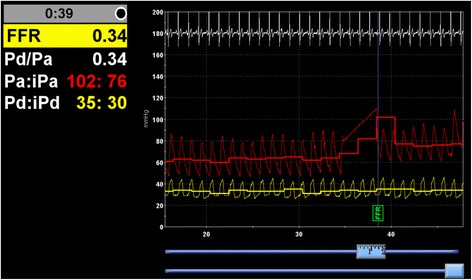
Fractional Flow Reserve (FFR) measurement of the right coronary artery lesion. A value of 0.34 was obtained, which is grossly below the cut-off value of 0.8

Fractional flow reserve (FFR) measurement in the RCA was strongly positive (Fig. 3) and the lesion was subsequently treated with a drug eluting stent. FFR measurement in the LAD was also physiologically positive and in turn this also treated with a single drug eluting stent. Following treatment he flew again with no recurrence of symptoms.

## Discussion

This gentleman had an unusual presenting history. His symptoms were assessed as likely cardiac (but not completely typical) and in the first instance a CT coronary angiogram was conducted. CT scans may over or under-estimate findings of coronary artery disease, meaning over, or under diagnosis. This was potentially an area of concern, in that his disease may have been missed. The findings on CT scanning correlated well with angiographic findings in this case. Given the nature of his symptoms, other methods of testing, such as stress echo or CMR, would have been valid alternatives at the time of his presentation. In November 2016, current National Institute of Clinical Excellence (NICE) guidelines on chest pain were reviewed, and coronary CT is now the initial investigation of choice in stable patients with typical or atypical chest pain [3].

### Physiological effects of high altitude

Commercial flights usually fly at an altitude of 25,000 to 35,000 ft. However, flight cabins are pressurised to an equivalent altitude of 5000–8000 ft., which equates to an inspired oxygen fraction of 0.17–0.15 at sea level [4]*.* Alveolar oxygen tension is known to decrease to 65 mmHg at 8000 ft., with a resultant reduction in arterial oxygen tension to 60 mmHg in healthy individuals [5]. Hypoxaemia augments the sympathetic nervous system and has many physiological effects. There is an increase in ventilatory effort in response to hypoxic conditions and the cardiovascular system responds by elevation of the heart rate, with subsequent reduction of stroke volume and an increased risk of angina and dysrhythmias in coronary patients [6]. The oxygen dissociation curves in healthy adults show that even at 65 mm. Hg the haemoglobin is still 80% saturated with oxygen [7]. The healthy adult is therefore hardly affected at the highest altitude that an aircraft cabin may reach in ordinary conditions, but an individual with impairment to the respiratory or cardiovascular system, may be distressed by this degree of hypoxia [8].

In one study, moderate altitude exposure in the elderly was associated with hypoxemia, sympathetic activation, and pulmonary hypertension resulting in a reduced exercise capacity that is predictable based on exercise performance at sea level. Patients with coronary artery disease who are well compensated at sea level do well at moderate altitude, although acute ischemia may be provoked at modestly lower myocardial and systemic work rates [9].

Cardiac output characteristically increases initially with hypoxia in a dose-dependent fashion, primarily due to tachycardia. The cardiac response slowly decreases over time despite continued hypoxia for unclear reasons [10, 11].

In our case described above, there was a critical right coronary artery stenosis, and a long segment of LAD disease, the severity of which was confirmed using fractional flow reserve. After coronary revascularisation, he made another journey from UK to Australia, and his symptoms did not recur. Therefore, we believe that the chest tightness which he had developed in-flight during his index journey was indeed due to cardiac ischaemia. The hypoxia during the flight would have led to an increased sympathetic response which brought on symptoms of ischaemia at rest. It remains unclear why he did not have any anginal symptoms on exertion prior to his flight. He led a rather sedentary lifestyle which may explain his lack of day-to-day symptoms.

### Current consensus on fitness to fly

A working group publication by the British Cardiovascular Society relating to fitness to fly for passengers with cardiovascular disease, was published in 2010 [12]. In patients with CCS class I-II angina, flight was unrestricted. Patients with class III angina are suggested to have airport assistance and in flight oxygen available. Delay of travel is recommended in patients with class IV angina. If there is no alternative to flight, a medical escort and in flight oxygen is advised. In the case of elective PCI with no complications, a rest period of 2 days is suggested.

In patients who have recently suffered an Non-ST segment elevation myocardial infarction (NSTEMI), or ST segment elevation myocardial infarction (STEMI), guidelines are based on age, whether reperfusion was successful, and ejection fraction after the event. Those aged less than 65 was successful reperfusion and an ejection fraction > 45% are deemed low risk, and can fly after 3 days. An ejection fraction > 40% with no symptoms of heart failure, and no urgently planned treatment or investigations are deemed medium risk, and are advised it is safe to fly after 10 days. Patients with an ejection < 40% with symptoms of heart failure and incomplete treatment or investigations are high risk, and advised to defer travel completely.

## Conclusion

This 68 year old gentleman had an index presentation of angina whilst on a commercial flight at cruising altitude. His underlying coronary artery disease was exacerbated by the combination of hypoxia, reflex tachycardia and increase in cardiac output plus paradoxical vasoconstriction [9]. After treatment, his symptoms did not recur on a further commercial flight.

## Abbreviations

CT: Computerised tomography
ECG: Electrocardiogram
FFR: Fractional flow reserve
LAD: Left anterior descending artery
LDL: Low density lipoprotein
NSTEMI: Non-ST segment elevation myocardial infarction
RCA: Right coronary artery
STEMI: ST segment elevation myocardial infarction
